# *In vitro *bioassay as a predictor of *in vivo *response

**DOI:** 10.1186/1742-4682-2-3

**Published:** 2005-02-07

**Authors:** Ross Barnard, Konstantin G Gurevich

**Affiliations:** 1 Department of Biochemistry, The University of Queensland, Brisbane, Qld 4072, Australia; 2 UNESCO Chair in healthy life for sustainable development, Moscow State University of Medicine and Dentistry, Delegatskay ulitsa, 20/1, 103473, Moscow, Russian Federation

## Abstract

**Background:**

There is a substantial discrepancy between *in vitro *and *in vivo *experiments. The purpose of the present work was development of a theoretical framework to enable improved prediction of *in vivo *response from *in vitro *bioassay results.

**Results:**

For dose-response curve reaches a plateau *in vitro *we demonstrated that the *in vivo *response has only one maximum. For biphasic patterns of biological response *in vitro *both the bimodal and biphasic *in vivo *responses might be observed.

**Conclusion:**

As the main result of this work we have demonstrated that *in vivo *responses might be predicted from dose-effect curves measured *in vitro*.

## Background

*In vitro *bioassay is very useful in biomedical experiments. It has the potential to yield very important data about molecular mechanism of action of any biologically active compounds. However, the major challenge for such experiments is extrapolation to *in vivo *responses. Unfortunately, there is a substantial discrepancy between *in vitro *and *in vivo *experiments, and there is a paucity of work directed to prediction of *in vivo *response from *in vitro *bioassay. So, the purpose of the present work was development of a theoretical framework to enable improved prediction of *in vivo *response from *in vitro *bioassay results.

## Results

A survey of literature revealed that most cases of dose-effect curves for *in vitro *experiments fall into three classes. They are:

• monophasic response;

• biphasic pattern;

• bimodal or polymodal dose-effect curve.

**MONOPHASIC RESPONSE **is the form most commonly reported in articles on *in vitro *bioassay. In these cases, with increasing dose of biologically active substance (BAS), the cellular response increases to a maximum (dose-response curve reaches a plateau). The most general schemes exhibiting this class of response can be classified as 3 classes:

(I) BAS regulation of enzyme activity,

(II) Ligand interaction with one type of receptor, and

(III) Ligand interaction with negatively cooperative receptors.

We will consider these three classes:

(I): BAS might regulate enzyme activity. It might be:

• substrate:

*E+S *←→ *ES *→ *E+P *→ *cell response*,     (scheme 1)

where *E *is enzyme, *S *is substrate, *ES *is enzyme-substrate complex, *P *is product. Cellular response is suggested to be proportional to product concentration.

Scheme (2) approximates the classic Michaelis scheme [1].

• enzyme activator (*A*)

*E+S *←→ *ES *→ *E+P *→ *cell response*

*E+A *←→ *EA *    (scheme 2)

*EA+S *←→ *EAS *→ *EA+P *→ *cell response increasing*,

Scheme (3) is characteristic of many BAS. The majority of these groups are vitamins and minerals, which are known to be enzyme cofactors and serve to increase enzyme activity.

• enzyme inhibitor (*I*)

*E+S *←→ *ES *→ *E+P *→ *cell response*

*E+I *←→ *EI *→ *no cell response*,     (scheme 3)

For example, there is the large class of drugs, whose action can be described with the help of scheme (4). This class is called "inhibitors of angiotensin-converting enzyme". These drugs are commonly used for hypertension treatment and prevention [2].

(II) Ligand interaction with one type of receptors:

*L+R *←→ *LR *→ *cell response *    (scheme 4)

where *L *is ligand (BAS), *R *is receptor, *LR *is ligand-receptor complex.

Scheme (4) is "classic" receptor theory as described by Clark (1937) [3].

For example, kinetic schemes of such type were proved in the case of estrogen regulation of gene expression [4], apolipoprotein AI, CII, B and E synthesis [5].

(III) Ligand interaction with negative cooperative receptors

*L+R *←→ *LR*

*L+LR *←→ *L_2_R *→ *cell response *    (5)

where *L_2_R *is complex ligand-receptor complexes.

Scheme (5) is characteristic for insulin receptors [6].

Kinetic equations for schemes (1)–(5) are well known [7]. They include "classic" Michaelis [1] and Clark [3] equations. It can be shown, due to the first order Taylor series, equations for the schemes (1)–(4) can be re-formulated from particle counter theory as:

*y *= B*x/(*1*+A*x)     (6)

and for scheme (5):

*y *= B*x^2^/(*1*+A*x^2^)     (7)

where *x *is incoming signal (*x *is BAS concentration). For scheme (1) *x *is substrate concentration, for scheme (2) it is activator concentration, for scheme (3) it is inhibitor concentration, for schemes (4) and (5) it is ligand concentration. *y *is cellular response for the *in vitro *system. *A *and *B *are scaling coefficients.

The BAS concentration in the whole organism changes as a function of time according to equation (14) (see Methods.) i.e.

*x(t) *= *C(t) *= *C_0_[exp(-k_el_γt)-exp(-k_1_t)] *    (8)

**Figure 1:**
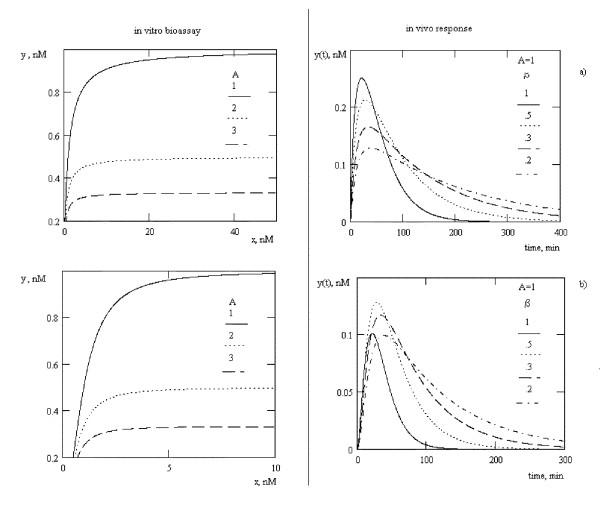
*In vivo *response for monophasic dose-effect curves measured *in vitro*. B = 1. a) equation (6), b) equation (7). *k*_*el *_= 0.0714 1/min, *k*_*1 *_= 0.0277 1/min, *C*_*0 *_= 1 nM, γ = β. Illustrative values for fig. 1, 2, 4 taken from Veldhuis et al., (1993) [8] and similar to those measured by Baumann et al., (1987)^9 ^for the clearance of growth hormone (GH).

We used equation (8) as the incoming signal, substituted this into equations (6) and (7) and solved analytically using Math Cad 8 graphing software (MathSoft Inc., Cambridge, MA, USA) to predict *in vivo *responses for monomodal *in vitro *dose-effect curves for schemes (1)–(5). We used illustrative values from works [8,9] and demonstrated that for such *in vitro *dose-effect curves, the *in vivo *response has only one maximum (fig. 1).

We define β (degree of conjugation) as the proportion of BAS that is free of binding proteins and is available to interact with cognate receptors. The larger is β, the larger the proportion of "free" BAS (see Methods). For equation (6) the value of this maximum is increasing as β increases; for equation (7) this value is maximum for mid-range β values.

### BIPHASIC PATTERNS OF BIOLOGICAL RESPONSE

In this case, in *in vitro *experiments the low doses of BAS stimulate cellular response, and the high doses inhibit it. So, a maximum is observed on the dose-response curve. The most common kinetic schemes for such response are:

• *Negative back loop *(substrate and product inhibition):

a) *E+S *←→ *ES *→ *E+P *→ *cellular response*

*ES+S *←→ *ES_2 _*    (9)

b) *E+S *←→ *ES *→ *E+P *→ *cellular response*

*ES *+ *P *↔ *ESP*

Such schemes are characteristic of glucose metabolism [1].

• Presence of two receptor types: one type stimulates cellular response, another type inhibits it.

*L+R *←→ *LR *→ *"positive" cellular response*

*L+R' *←→ *LR' *→ *"negative" cellular response *    (10)

where *R *are receptors of the first type, *R' *are receptors of the second type, *LR*, *LR*' are ligand-receptor complexes with different receptor types.

This mechanism has been proven for estrogen regulation of nitric oxide synthase (activity in the rat aorta [10]; protein pS2 expression in hormone-dependent tumors [11] and so on.

• Desensitization of cellular receptors

*L+R *←→ *LR *→ *positive cellular response*

*LR *→ *decrease in receptor number *    (11)

It has been suggested, that mechanism (11) is basic for drug tolerance [7]. For example, this mechanism was described for uretal cell stimulation by 17-β-estradiol. Before estradiol treatment, expression of estrogen receptors mRNA in cells was much higher then after 12-days estradiol administration [12]. It is well known that endogenous opioid receptors become down regulated after chronic exposure to exogenous opioids [13] and receptor down-regulation has often been observed to follow acute exposure to hormones including growth hormone [14].

• Change of effector's molecule conformation:

*"Active" conformation *+ *ligand suplus *←→ *"Passive" conformation *    (12)

Scheme (12) was suggested by Bootman and Lipp (1999) [15] for Ca^++ ^regulation of 1,4,5-trisphosphate activity. The authors suggested that Ca^++ ^surplus induces a change in Ca^++^-channel conformation from "open" or "active" to "closed" or "passive" [15].

For schemes (9)–(12), due to the first order Taylor series, this kinetic equation can be derived:

*y *= A*x*exp(-B*x)     (13)

**Figure 2:**
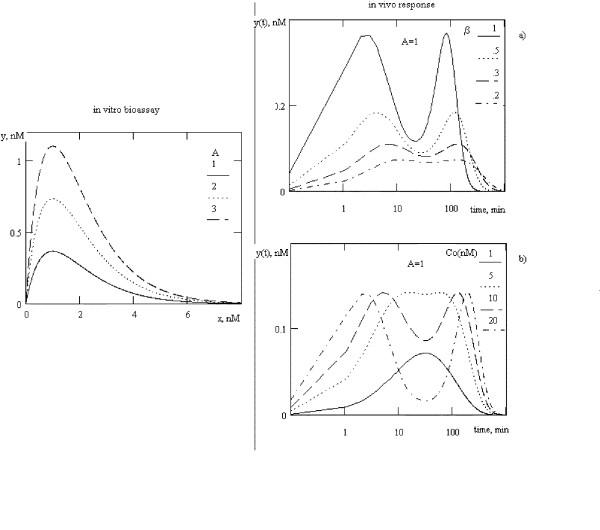
*In vivo *response for biphasic dose-effect curves measured *in vitro*. B = 1. a) variation of β, C_0 _= 1 nM, b) variation of C_0_, β = .388. *k*_*el *_= 0.0714 1/min, *k*_*1 *_= 0.0277 1/min, γ = β.

Using equation (13), we obtained a prediction of *in vivo *biphasic dose-effects curves (fig. 2). As is apparent from the figure, the magnitude and the analytical appearance of *in vivo *response is affected by the dose of BAS and its degree of conjugation (β). Both the bimodal and biphasic *in vivo *responses might be observed for biphasic dose-effect curves. Changes of dose of BAS concentration or its conjugation with blood proteins (or their concentration) might dramatically change the form of *in vivo *response. For the simulations shown in Figure 2 we used values for *k*_*el *_and *k*_*1 *_and blood volume (4.9 liters) based on measurements by Baumann et al. (1987) [9] and Veldhuis et al. (1993) [8] for growth hormone secretion, clearance and pulsatility. Polymodal biological responses are commonly observed in biological systems. It has been demonstrated, that in some experimental systems, administration of a single, bolus dose of hormone produces a polymodal response [16].

**Figure 3:**
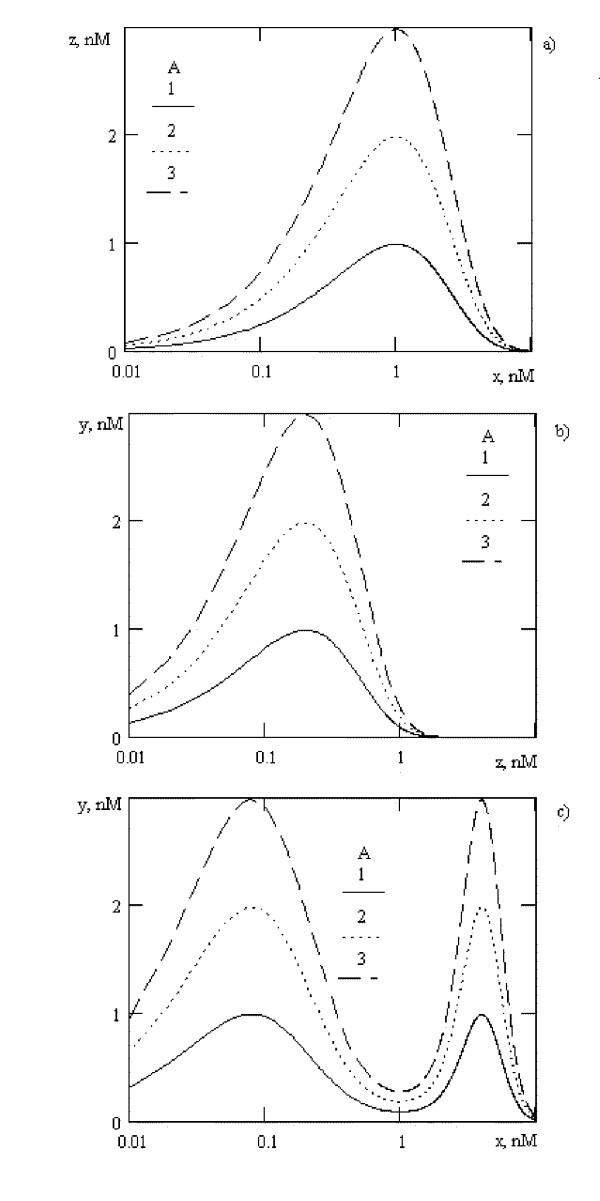
Possible mechanism of bimodal dose-effect curve formation for *in vitro *systems. a) intermediate *z *formation as function of *x *concentration, *B*_*1 *_= 1, b) final product *y *formation as function of *z *concentration, *B*_*2 *_= 5, c)summary dose-response curve. See comments in the text of the article.

Bimodal dose-effect curves are usually observed for BAS with regulatory activity [17,18]. The mechanism of their formation is still unclear. From our point of view, bimodal dose-response curve might be described by superposition of two biphasic dose-effect curves with different *B *value. This might be observed in cascade system of signal transduction and amplification. If *x *regulate intermediate *z *formation in biphasic way with *B*_*1*_, and *z *has biphasic response on *y *formation with *B*_*2*_, then if *B_1_*<*B_2_*, summary dose-effect curve (*y *concentration from *x*) is bimodal (fig. 3). Differences in *B*_*1 *_and *B*_*2 *_value define the maximum points. For example, with *B*_*2 *_increasing, the interpeak distance will also increase.

**Figure 4:**
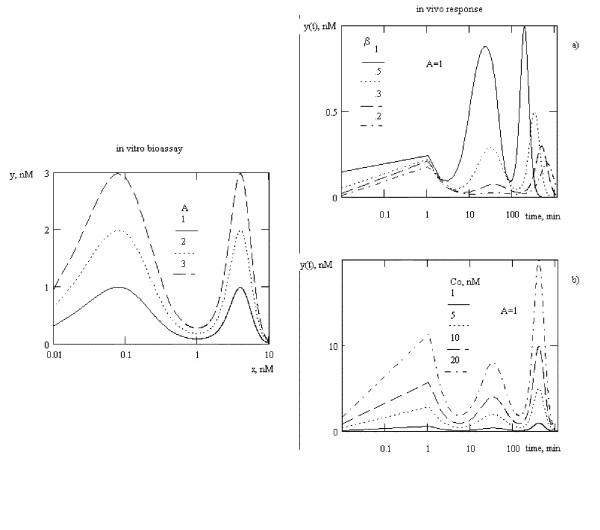
*In vivo *response for bimodal dose-effect curves measured *in vitro*. *B*_*1 *_= *1*, *B*_*2 *_= *5*. a) variation of β, C_0 _= 1 nM, b) variation of C_0_, β = .388. *k*_*el *_= 0.0714 1/min, *k*_*1 *_= 0.0277 1/min, γ = β.

For systems, which have bimodal dose-effect curve *in vitro*, the polymodal response *in vivo *is observed (fig. 4). The form of this response might be change to "seems constant" due to BAS concentration of β value. The differences of maximum values are observed, this differences is time-dependent: the highest maximum is observed with the longest observation. It might be demonstrated, that with change of *B*_*2 *_value to 20, only bimodal *in vivo *response will be observed. So, the form and the value of maximums are dependent from the dose of BAS and degree of conjugation.

## Discussion

Analogues of hormones are commonly used in medicine for hormone replacement therapy (for example in post-menopausal women), for oral contraception, as anabolic drugs, for asthma therapy and so on [2]. But engineered modifications of hormones, growth factors or their analogs are likely to differ from the native analogues in their affinity for binding proteins. In view of this, an important practical consequence of our simulations results are that the testing of newly designed hormones in *in vivo *systems (with endogenous binding proteins) will require measurements of acute biological response at multiple concentration and time points. For longer-term responses requiring protein synthesis (such as a secretion of body mass or longitudinal bone growth), it could be argued that such multiple time point studies would not be as important. However, in so far as long term biological responses are the consequence of critical initial events which may require threshold concentrations of free hormone, or repeated patterns of hormone exposure over prolonged periods [16,19], this assumption may not be justified.

Another application of our work may be the study of hormone functions in glandular tumour disorders. With these disorders, there is usually serious metabolic or hormonal dysfunction. From our point of view, it may be not only due to gland biosynthesis of abnormal hormone. Tumour-produced hormones may not differ structurally from their normal analogues. The dysfunctional occurs due to abnormal concentrations of hormones, which are synthesised by tumours. As it follows from our results, changes in concentrations can dramatically change the form and value of biological response. On the other hand, in many tumour disorders the concentrations of binding proteins are changed. For example, in ovarian carcinoma the changes of sex binding protein and ratio free/bound sex hormones (β) are observed [20]. As follows from our results, this can dramatically change the biological response to such hormones, i.e. apparent biological functions. So with testing *in vitro *such hormones seems to be normal (and they may be normal), but *in vivo *they may have abnormal effects due to changes of their binding protein concentration, or ratio free/bound hormone.

## Conclusion

So, as a result of this work we have demonstrated that *in vivo *responses might be predicted from dose-effect curves measured *in vitro*. For monophasic curves, *in vivo *response is proportional to BAS concentration. For the most complex *in vitro *curves, the value and the form of *in vivo *response depends in a predictable way on the dose of BAS and its degree of conjugation.

## Methods

To obtain the discussed results we used linear pharmacokinetics model:



where: *m*_*1*_*(t) *mass of biologically active substance (BAS) in the place of infusion, *m*_*2*_*(t) *mass of BAS in compartment (blood), *k*_*1*_,*k*_*el *_constants of hormone diffusion from place of infusion to blood and excretion form blood (accordingly).

Many of biologically active substances are conjugate into complexes with blood proteins (for example: GH, nerves growth factor, IGF-1):

*B+P *⇔^*K *^*HP *    (15)

where *B *is BAS, *P *is blood protein, *BP *is BAS-protein complex, *K *is dissociation constant.

For many BAS, concentration of free (not bound with blood proteins) BAS is equal to:

*[B] ≈β [B_0_] *    (16)

where β is constant ("degree of conjugation"), [*B*] is concentration of free BAS, [*B*_0_] is initial concentration of BAS. If β = *1 *then BAS dose not conjugate with protein. If β = *0 *then all BAS is in conjugate form.

It may be that only conjugate BAS (for example, bilirubin), or only unconjugated BAS can be excreted form the blood (for example, sex hormones). This means that for scheme (14) the law of mass action will be written in the next way:

*dm_1_/dt *= -*k_1_m_1_*, *m*_*1*_(0) = *M*

*dm_2_/dt *= *k_1_m_1 _*- γ*k_el_m_2_*, *m_2_(0) = 0 *    (17)

where γ is a constant. γ = 1-β if only conjugate form of BAS can be excreted and γ = β if only unconjugated form is excreted.

But γ is a constant with respect to *t*: γ = *const(t)*. This means that solution of system (17) is:

*C(t) *= *C_0_[exp(-k_elγ_t)-exp(-k_1_t)] *    (18)

where *C(t) *is BAS concentration in the blood compartment (*C *= *m*_*2*_/*V*, *V *= const (about 5 liters) is blood volume), *C*_*0 *_is seems initial BAS concentration (*C*_*0 *_= *M/V*).
